# Neurodegenerative Disorders of the Eye and of the Brain: A Perspective on Their Fluid-Dynamical Connections and the Potential of Mechanism-Driven Modeling

**DOI:** 10.3389/fnins.2020.566428

**Published:** 2020-11-12

**Authors:** Giovanna Guidoboni, Riccardo Sacco, Marcela Szopos, Lorenzo Sala, Alice Chandra Verticchio Vercellin, Brent Siesky, Alon Harris

**Affiliations:** ^1^Department of Electrical Engineering and Computer Science, Department of Mathematics, University of Missouri, Columbia, MO, United States; ^2^Department of Mathematics, Politecnico di Milano, Milan, Italy; ^3^Université de Paris, CNRS, MAP5 UMR 8145, Paris, France; ^4^Inria Paris, Paris, France; ^5^IRCCS - Fondazione Bietti, Rome, Italy; ^6^Department of Ophthalmology, Icahn School of Medicine at Mount Sinai, New York, NY, United States; ^7^Department of Ophthalmology, University of Pavia, Pavia, Italy

**Keywords:** neurodegenerative disorders, fluid-dynamics, mathematical modeling, glaucoma, intraocular pressure, cerebrospinal fluid pressure

## Abstract

Neurodegenerative disorders (NDD) such as Alzheimer's and Parkinson's diseases are significant causes of morbidity and mortality worldwide. The pathophysiology of NDD is still debated, and there is an urgent need to understand the mechanisms behind the onset and progression of these heterogenous diseases. The eye represents a unique window to the brain that can be easily assessed via non-invasive ocular imaging. As such, ocular measurements have been recently considered as potential sources of biomarkers for the early detection and management of NDD. However, the current use of ocular biomarkers in the clinical management of NDD patients is particularly challenging. Specifically, many ocular biomarkers are influenced by local and systemic factors that exhibit significant variation among individuals. In addition, there is a lack of methodology available for interpreting the outcomes of ocular examinations in NDD. Recently, mathematical modeling has emerged as an important tool capable of shedding light on the pathophysiology of multifactorial diseases and enhancing analysis and interpretation of clinical results. In this article, we review and discuss the clinical evidence of the relationship between NDD in the brain and in the eye and explore the potential use of mathematical modeling to facilitate NDD diagnosis and management based upon ocular biomarkers.

## 1. Introduction

Neurodegenerative disorders (NDD) represent a heterogeneous group of diseases characterized by progressive structural and functional degeneration of the central and peripheral nervous systems. NDD, such as vascular dementia, Alzheimer disease (AD), Parkinson's disease (PD), and Huntington's disease (HD), are common causes of morbidity and mortality worldwide, especially in the elderly population (Erkkinen et al., 2018). Other neurodegenerative pathologies affecting the elderly such as glaucomatous optic neuropathy greatly reduce a person's quality of life. As life expectancy continues to increase, it is critical to elucidate the pathophysiology of NDD in order to improve diagnostic and therapeutic options and reduce the ever-increasing burdens NDD place on the health care system.

Thanks to its special connection to the brain and its accessibility to measurements, the eye provides a unique window on the brain, thereby offering non-invasive access to a large set of potential biomarkers that might help in the early diagnosis and clinical care of NDD. However, characterizing ocular biomarkers as surrogates of the brain status is far from trivial. Clinical measurements are influenced by many factors that vary among individuals, such as blood pressure, intraocular pressure (IOP), and cerebrospinal fluid (CSF) pressure. Furthermore, some of these factors may be systemic (e.g., blood pressure, CSF pressure) or local to the eye (e.g., IOP), thereby making it extremely difficult to isolate their contributions to pathogenic processes in *in vivo* settings. Thus, despite their accessibility to noninvasive imaging, ocular measurements remain challenging to interpret in view of NDD biomarkers due to the lack of quantitative methods to identify the most relevant pathogenic mechanisms in specific patients.

In the last decades, mechanism-driven modeling has emerged as a valuable complement to statistical methods in analyzing and interpreting the outcomes of clinical and experimental studies (Sacco et al., 2019a). The purpose of mechanism-driven models is to translate concepts of physics and physiology into mathematical equations that can be solved on a computer, thereby providing a sort of virtual laboratory where multiple scenarios can be simulated and hypotheses can be tested. Ultimately, mechanism-driven models can provide insights on the fundamental physiological mechanisms at play in complex multifactorial diseases and aid the design of new clinical and experimental studies.

The main goal of this article is to look at NDD from a broad multidisciplinary perspective, where clinical evidences of NDD in the brain and in the eye are reviewed (section 2) and critically discussed (section 3), with particular focus on fluid-dynamical aspects. Mechanism-driven models are presented in the context of other methods for data analysis (section 4), followed by an overview of currently available models for the eye and their connection to the brain (section 5). The potential of utilizing mechanism-driven models as virtual laboratories is illustrated by means of a specific example involving the interplay between IOP and CSF pressure on the biomechanics and hemodynamics of the optic nerve head, which is an anatomical site of particular interest for the eye-brain connection (section 6). Finally, the article concludes with an outlook on how to further develop this multidisciplinary approach in order to enable NDD diagnosis and monitoring via noninvasive ocular measurements (section 7).

## 2. Overview of Clinical Evidence

While the etiology of NDD is not fully understood, many studies have shown that alterations in brain fluid balance including cerebral blood flow and CSF flow often precede cognitive damage (Weller et al., 2009; Sagare et al., 2013). For example, impaired blood supply to the brain and subsequent hypoperfusion have been shown to be involved in the pathogenesis of vascular dementia and AD (Esiri et al., 1999; Jellinger and Attems, 2007; Jellinger, 2008). Specifically, AD is associated with ineffective drainage of interstitial fluid leading to an abnormal accumulation of amyloid-β (Aβ) in the perivascular space (Weller et al., 2009) and decreased levels of Aβ in the CSF (Blennow et al., 2006; Shaw et al., 2011; Lehmann et al., 2014). Several studies have shown chronic cerebral hypoperfusion involvement in the progression of cognitive decline in AD patients (Lin et al., 2011; Shang et al., 2016; Zhai et al., 2016; Shi et al., 2019), and an impairment of Aβ clearance has been suggested to play a primary role in the pathophysiology of the disease (Gallina et al., 2015). Alongside AD, alterations in cerebral blood flow have been implicated in the pathogenesis of Parkinson's disease (Hirano et al., 2012; Heron et al., 2014). In addition, patients with amnestic mild cognitive impairment present abnormally high mean total cerebral arterial flow, whereas patients with idiopathic normal pressure hydrocephalus present abnormally high aqueductal CSF oscillations (Sankari et al., 2011). Impaired homeostatic brain fluid balance and clearance therefore appear to represent a key component of a wide spectrum of NDD.

The eye is directly linked to the brain via the optic nerve. Progressive neurodegeneration of the optic nerve and corresponding death of the retinal ganglion cells occurs in glaucoma, a leading cause of irreversible blindness worldwide. Elevated IOP is a well-known risk factor for onset and progression of the disease, but other systemic fluid-balanced biomarkers, such as blood pressure, ocular perfusion pressure, and CSF pressure have been implicated in the disease process (Berdahl et al., 2008; Caprioli and Coleman, 2010; Harris et al., 2019, 2020). Reduced ocular blood flow and higher vascular resistance have been shown to occur with age with subtle differences in men and women and, along with senescence, may contribute to increased risk in older age (Harris et al., 2000). Decreased cerebral blood flow and diffuse cerebral ischemic changes as well as impaired vascular autoregulation have all been identified in glaucoma patient populations (Harris et al., 2003, 2007, 2013). While traditionally considered a disease limited to the eye, current understanding identifies glaucoma as a neurodegenerative disorder (Gauthier and Liu, 2016), as it shares several physiological similarities with other NDD, such as AD and PD.

Neurodegeneration of the eye and brain tissues, fluid homeostasis, and progressive NDD disease processes therefore have significant interconnectivity. Several studies have shown that different regions of the central nervous system, and not only the optic nerve, are in fact affected by glaucomatous damage, i.e., the intracranial optic nerves, the lateral geniculate nucleus, and the visual cortex (Gupta et al., 2006, 2009). Specifically, glaucoma and the other NDD share common pathophysiologic mechanisms, such as the programmed cell death (apoptosis) of specific groups of neurons, and the diseases' spread via trans-synaptic degeneration (Gupta and Yücel, 2007). In addition, neuroinflammation and microglial activation are commonly reported pathogenetic features in glaucoma and other NDD, such as AD and PD (Ramirez et al., 2017). Both aging and a positive family history are common risk factors for all the aforementioned diseases that are characterized by insidious onset (Gauthier and Liu, 2016). Mancino et al. (2018) recently suggested that glaucoma and AD can be considered as two manifestations of one singular age-related NDD of the brain. An alteration in the level of the intracranial pressure has been suggested as a possible common mechanism between glaucoma and AD. In fact, lower levels of CSF pressure have been shown in both glaucomatous patients (Berdahl et al., 2008) and AD patients (Silverberg et al., 2006). Another indication of the cerebral involvement in the glaucomatous damage has been shown by Mercieca et al. (2016), who found an increased number of cerebral small vessel disease assessed by magnetic resonance in patients with glaucoma, compared to healthy controls.

**Figure 1:**
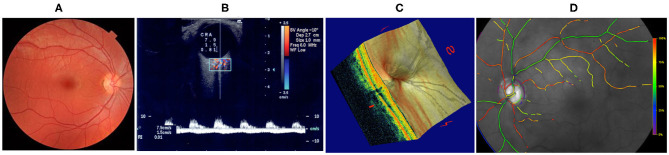
Techniques for non-invasive ocular measurements (Guidoboni et al., 2013). **(A)** Fundus camera; **(B)** Color Doppler Imaging; **(C)** Fourier-Domain Optical Coherence Tomography (FD-OCT); **(D)** Retinal Oximetry. Images reproduced from Guidoboni et al. (2013), with permission.

Vascular changes in the brain have also been associated with vascular changes in the eye (Heringa et al., 2013; Jindal, 2015). This is not surprising since, in many aspects, the eye is a protrusion of the brain. The blood supply to the eye is secured by the ophthalmic artery, which is a branch of the internal carotid artery; the blood drains from the eye into the cavernous sinus, which also receives blood from superficial cortical veins; the CSF fills the subarachnoid space which surrounds the brain and extends all around the optic nerve (Fleishman and Berdahl, 2019; Prada et al., 2019). In addition, the retina shares anatomic, embryologic, and physiologic characteristics with the cerebral microvasculature and the neurons of the central nervous system (Heringa et al., 2013; London et al., 2013). Indeed the eye is the only place in the human body where structural and functional vascular features can be observed and measured easily and non-invasively down to the capillary level (Harris et al., 2003; Weinreb and Harris, 2009). Fundus camera gives images of the retinal vasculature, color Doppler imaging measures velocity profiles in the main arteries supplying the eye, Heidelberg retinal flowmeter analyzes the hemodynamics in the retinal microcirculation, retinal oximetry gives oxygen levels in the arterioles and venules of the retina, and optical coherence tomography (OCT) and OCT angiography (OCTA) capture high-resolution information of the ocular structure combined with measurements of the retinal vessels in the optic nerve head and macula regions (Weinreb and Harris, 2009; Guidoboni et al., 2013; Kashani et al., 2017; Gross and Prada, 2019), see Figure 1. Given the relationship between eye and brain, current research to detect signs on neurologic disorders via non-invasive imaging modalities at the ocular level is highly suggested.

Research focused on using the eye as a window into the brain is in its juvenile stage, yet several promising findings have emerged in pilot studies. Specifically, patients suffering from NDD and several other neurological diseases often display noticeable vascular and structural alterations in the eye. Reed et al. (2017) showed how early signs of AD may be detected at the level of the eye as retinal synaptic dysfunction and highlighted the advantages of analyzing the retina structure and function via non-invasive imaging techniques for earlier detection of AD. AD has been associated with reduced cerebral and ocular blood flow, retinal vascular attenuation, increased standard deviation of retinal vessel widths, reduced complexity of the retinal vascular branching pattern and reduced tortuosity of retinal venules (Langham, 2009; Frost et al., 2013; Li et al., 2014). Multiple small sample studies have shown thinning of the retinal nerve fiber layer in patients with AD (Doustar et al., 2017). In a recent study from O'Bryhim et al. (2018), subjects with preclinical AD showed retinal microvascular and structural anomalies assessed via OCTA. PD is also associated with retinal thinning (Satue et al., 2013; Garcia-Martin et al., 2014) which, in turn, is associated with reduced retinal blood flow (Januleviciene et al., 2008). Over a 5 year follow-up period, greater changes in the retinal nerve fiber layer and macular thickness were found in patients with PD compared to controls (Satue et al., 2017). Also, PD progression was associated with the structural changes of the retinal nerve fiber layer (Satue et al., 2017). A correlation between disease severity and structural changes in the retinal nerve fiber layer thickness and macular volume was also found in patients with HD (Kersten et al., 2015). Patients with multiple sclerosis have also shown a reduction in the optic nerve head perfusion, and in the thickness of the peripapillary retinal nerve fiber layer and macular ganglion cell complex compared to healthy subjects (Spain et al., 2018). Finally, the space flight-associated neuro-ocular syndrome (SANS) is another example of disease with ocular and cerebral manifestations. This term indicates the spectrum of clinical signs found in astronauts undergoing long duration space flight, such as optic disc swelling, choroidal, and retinal folds, flattening of the ocular globe, hyperopic shift in refraction, and infarcts of the nerve fiber layer (Mader et al., 2019). The pathogenesis of the disease is not fully understood, and imaging modalities such as OCTA present a great potential in order to non invasively study the structural changes that occur in astronauts (Lee et al., 2018).

## 3. Open Questions and Matters of Debate

The eye and its anatomical and fluid-based connectivity to brain tissues, along with its accessibility to imaging measurements, provides a unique window for non-invasive biomarkers that might help in the early diagnosis and clinical care of NDD. The scientific community is actively investigating how to use vascular and CSF-related biomarkers for NDD suspects, since early diagnosis significantly reduces negative outcomes in NDD treatment (Zimmermann et al., 2011; Corvol, 2012). However, clinical measurements are influenced by many factors that vary among individuals and cannot be isolated *in vivo*, thereby posing serious challenges for the interpretation of such measurements both for neurologic and ocular diseases. For example, despite the fact that the relevance of CSF biomarkers for AD diagnosis is clearly established, the question remains on how to use these data since not all biomarkers are always pathologic (Lehmann et al., 2014). Also, the pathogenesis of AD is still debated, and further research is needed to understand the relative importance of blood and CSF flows in Aβ clearance (Sagare et al., 2013), and the roles of excessive production or inefficient removal in Aβ abnormal accumulation (Weller et al., 2009). Similar considerations hold true for ocular biomarkers. Specifically, even though retinal biomarkers have been shown to hold great potential for early AD detection (Grimaldi et al., 2018), they still need validation in order to be integrated in the clinical practice as translatable tools for screening NDD (Alber et al., 2020). Studies are also needed to further the understanding of the interplay of different ocular (IOP, ocular perfusion pressure), and systemic (blood pressure, CSF pressure) parameters in the pathophysiology of the disease (Caprioli and Coleman, 2010; Jonas et al., 2015). Finally, the distribution of a drug within the brain involves complex mechanisms, influenced by different factors including those involved in transport across the blood-brain barrier and drug target binding within the brain. Importantly, Vendel et al. (2019), demonstrated how three-dimensional mathematical models can elucidate drug pharmacology that act at the level of the central nervous system, by integrating physiologic parameters that influence drug distribution within brain tissues. Therefore, improved methods for data analysis via mathematical modeling would be critically important to further the understanding of the role of different factors in the pathogenesis of brain and ocular NDD and diseases, thus ameliorating the available diagnostic and therapeutic options and the quality of life of million of patients worldwide.

## 4. Methods for Data Analysis: Role of Mechanism-Driven Modeling

The challenge of characterizing the role of individual risk factors in the pathogenesis of multifactorial diseases, such as NDD, is ubiquitous in medicine. Oftentimes, different factors interact with each other and may increase or decrease the overall disease risk for a given individual, leaving clinicians with the dilemma of how to select the best therapeutic approach for each single patient. Ultimately, the quest of deciphering how various factors contribute to the disease pathogenesis in each patient is what drives *precision medicine*, from which the management of NDD would greatly benefit.

Typical methodological approaches that are utilized to solve the puzzle of multifactorial diseases include the acquisition of data in animals and in humans. Animal studies have the advantage that they allow for controlled laboratory protocols and extremely invasive procedures, but are limited by the extent to which a disease process occurring in the animal model is representative of what occurs in humans. Human studies have the advantage that they provide information on the disease process as it happens on the subject of interest, but are limited by the type of procedures that can be performed and the type of data that can be acquired.

The data acquired in clinical and experimental studies have been traditionally analyzed using statistical and computational methods, giving rise to whole new scientific fields such as biostatistics and bioinformatics. Artificial intelligence (AI) is emerging as a powerful tool to connect the dots among different types of data (e.g., medical images, demographic information, medications, medical history) acquired on different types of subjects (e.g., humans and animals) (Jiang et al., 2017). For example, AI is enabling unprecedented automated analysis of images of the eye (Schmidt-Erfurth et al., 2018; Ting et al., 2019) and the brain (Akkus et al., 2017; Lee et al., 2017). However, to date, it remains very challenging to interpret the outcomes of AI algorithms on the basis of physiological mechanisms that may be most relevant in the disease process of a specific patient.

A complementary approach is offered by mechanism-driven modeling, where fundamental principles of biophysics and physiology are combined and translated into mathematical equations that can be used as virtual laboratories to simulate numerous “*what if”* scenarios at minimal cost (Sacco et al., 2019b). Animal and human data are essential to develop mechanism-driven models and characterize the values of model parameters. Mechanism-driven models can then be used to test hypotheses, formulate new conjectures and inform the design of new experiments. Recently, the scientific community is moving toward combining mechanism-driven models and AI algorithms, in order to improve the explainability of AI outcomes (Baker et al., 2018). Thus, mechanism-driven models serve as valuable complements of experimental and clinical research and may help disentangle the role that different factors play in complex diseases, such as NDD.

Combining mechanism-driven modeling and imaging in a unified computational framework has proved to be a powerful and successful approach in the domain of cardiovascular disease research, surgical planning, and diagnostics in the last years. There is particular promise for elucidating the complex interplay between hemodynamics and biomechanics from this emerging area (Taylor and Figueroa, 2009; Niederer et al., 2019). Examples of key applications include treatment and monitoring of cardiovascular conditions (Vignon-Clementel et al., 2010), medical device evaluation (Chen et al., 2006), and non-invasive diagnostics and surgical planning (van Bakel et al., 2018). In the following sections, we will focus on mechanism-driven models for the eye, in the view of their connection to the brain.

## 5. Overview of Mechanism-Driven Models of the Eye and Their Connection to the Brain

Mechanism-driven models have been proposed for the study of various aspects of ocular biophysics. Biomechanical and fluid-dynamical aspects are of particular relevance when viewed in the perspective of the eye-brain connections. Biomechanical studies have the goal of predicting distributions of stresses and strains that originate in the tissues as a result of specific challenges, such as those experienced during diagnostic or therapeutic procedures or pathological changes in IOP or intracranial pressure (ICP) (Roberts and Liu, 2017; Roberts et al., 2018). Fluid-dynamical studies have the goal of predicting distributions of pressures and velocities characterizing the flow of a fluid in the tissue. In the eye, such studies have focused on the flow of blood, aqueous humor and tear film, with few contributions considering the dynamics of vitreous humor and cerebrospinal fluid, as reviewed in recent works (Arciero et al., 2019; Braun et al., 2019; Dvoriashyna et al., 2019; Repetto and Dvoriashyna, 2019; Sala et al., 2019b).

It is important to emphasize that, in response to a challenge, the tissue may exhibit both biomechanical and fluid-dynamical responses, whose behavior is coupled rather than disjoint. For example, when IOP is elevated, ocular tissues may undergo larger deformations (biomechanical response) and, as a consequence, blood vessels may be distorted or compressed, thereby compromising the blood flow through the tissue (fluid-dynamical response). Furthermore, sustained biomechanical and fluid-dynamical alterations will impact the delivery of oxygen and nutrients to the tissue and the removal of metabolic products, such as Aβ, from the tissue, and will likely induce a cascade of remodeling processes which, in turn, will alter the way in which the tissue will respond to future challenges. The development of mechanism-driven models to study the complex interaction between biomechanical and fluid-dynamical responses requires multiscale and multiphysics modeling techniques, which are still active areas of research in mathematical and computational sciences (Sacco et al., 2019b). As a result, only few mechanism-driven models are currently available to study the interaction among diverse biophysical aspects of ocular physiology. Examples include models coupling biomechanics and hemodynamics (Guidoboni et al., 2014a,b,c; Arciero et al., 2019; Sala, 2019) and models coupling hemodynamics and oxygenation (Arciero et al., 2013, 2019; Causin et al., 2016; Carichino et al., 2016; Fry et al., 2018). Most of these models have focused on macroscale phenomena, with only few models accounting for their counterparts at levels of single cells and ion exchangers on the cellular membrane (Mauri et al., 2016; Sala et al., 2019a; Sacco et al., 2020). As a further note, to date, only few mechanism-driven models have included the dynamics of Aβ (Craft et al., 2002; Puri and Li, 2010; Das et al., 2011; Kyrtsos and Baras, 2015; Franchi and Lorenzani, 2016, 2017). However, these models have been developed in the context of the brain and their applicability to the eye remains to be assessed.

Biomechanical and fluid-dynamical aspects play a very important role in the multifaceted connection between eye and brain. The blood to the eye is supplied by the ophthalmic artery, which branches from the internal carotid artery on its way to the brain. The ocular venous blood drains into the facial vein, the pterygoid venous plexus and the cavernous sinus, the latter being one of sinuses collecting also cerebral venous blood (Prada et al., 2019). The cerebrospinal fluid that occupies the subarachnoid space between the arachnoid mater and the pia mater in the brain while flowing through the ventricular system and the spinal cord, also fills the subarachnoid space around the optic nerve (Fleishman and Berdahl, 2019). Interestingly, both eye and brain are characterized by internal pressures, namely intraocular pressure (IOP) and intracranial pressure (ICP), that are exerted by fluids on the surrounding tissues. Cerebrospinal and interstitial fluids are the main contributors to establishing ICP in the brain (Fleishman and Berdahl, 2019); in the eye, IOP is mainly due to the balance between production and drainage of aqueous humor, which is the clear fluid filling the anterior chamber between the lens and the cornea (Toris et al., 2019).

The dynamics of blood, aqueous humor, and CSF in the eye and in the brain are intrinsically intertwined, with alterations in their flow rates and pressures being associated with several pathological conditions, including glaucoma, NDD and SANS, as discussed in section 2. The study of this complex system via mechanism-driven models is still in its infancy, with most investigations being motivated by research in glaucoma (Carichino et al., 2014; Harris et al., 2019; Sala, 2019; Sala et al., 2019b) and SANS (Nelson et al., 2017; Feola et al., 2018; Kaskar et al., 2019; Salerni et al., 2019).

In the following section, we illustrate by means of an example how mechanism-driven models can be used to gain insights on the biomechanics and hemodynamics in the eye and their role in the eye-brain connection.

## 6. Utilizing Mechanism-Driven Models as Virtual Laboratories: a Specific Example

As a specific example, in this section we will illustrate how mechanism-driven models can help understand and quantify the consequences that different levels of IOP and CSF pressure have on ocular biomechanics and hemodynamics. Such an example is particularly relevant in view of utilizing ocular measurements for NDD diagnosis and monitoring. CSF changes have been associated with disease conditions, along with biomechanical and hemodynamical changes in the eye, as discussed in section 2. However, ocular measurements in the eye are also likely to be affected by conditions that are local to the eye, such as the level of IOP. Thus, in order to devise effective ocular biomarkers for NDD, we should be able to tell how a specific condition in the brain, e.g., altered CSF pressure, is going to manifest in the eye of a specific individual, e.g., having a IOP of 17 mmHg rather than 11 mmHg.

**Figure 2:**
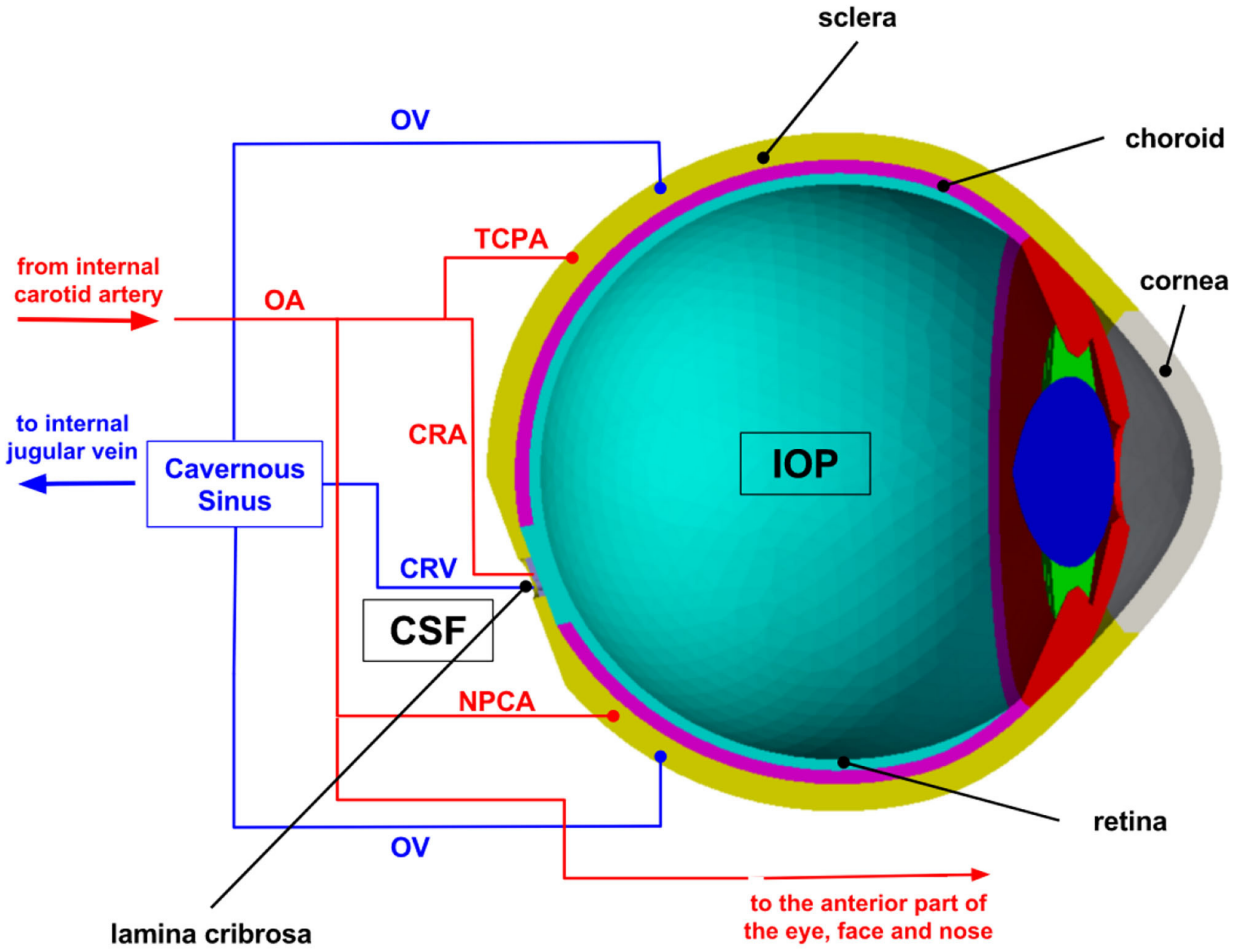
Schematic overview of the multiscale modeling framework implemented in the Ocular Mathematical Virtual Simulator (OMVS). Image reproduced from Sala et al. (2018a), with permission. OA, ophthalmic artery; OV, ophthalmic vein; CRA, central retinal artery; CRV, central retinal vein; NPCA, nasal posterior ciliary artery; TPCA, temporal posterior ciliary artery; IOP, intraocular pressure; CSF, cerebrospinal fluid.

The mechanism-driven model discussed in this section has been published in Sala (2019) and Sala et al. (2018a,b) and includes several anatomical components of the eye, as schematized in Figure 2. In particular, the lamina cribrosa is a collagenous structure located within the optic nerve head that bears remarkable relevance for the connection between eye and brain. The lamina cribrosa facilitates the passage of axon bundles transmitting the neural signal from the retina to the brain and allows the central retinal artery and vein to perfuse the retina, while helping maintain the pressure difference between the intraocular space and the optic nerve. The pressure in the intraocular space is the IOP and is primarily due to the aqueous humor filling the anterior chamber (Toris et al., 2019), whereas the pressure in the optic nerve is often referred to as retrolaminar tissue pressure (RLTp) and is primarily due to the CSF filling the subarachnoid space (Morgan et al., 1995, 1998). Healthy baseline values of IOP and RLTp are difficult to definitely set for all persons of different ages, genders, races, and in consideration of co-morbidities. Reported values of approximately 15 and 7 mmHg, respectively, may represent reasonable baseline choices (Harris et al., 2020). The difference between IOP and RLTp is often referred to as translaminar pressure difference (TLPd), whose alterations have been associated with glaucoma, papilledema and SANS (Marek et al., 2013; Mader et al., 2019). However, many questions remain unanswered, including: (i) whether and to what extent individual levels of IOP and RLTp, in addition to their difference, contribute to originate pathogenic processes; and (ii) whether and to what extent changes in IOP, RLTp, and TLPd induce altered conditions in biomechanics and/or hemodynamics within the lamina cribrosa.

To help address these questions, we utilize a mechanism-driven model, also called Ocular Mathematical Virtual Simulator (OMVS), that allows the user to simulate the blood circulation in the central retinal vessels and the lamina cribrosa (hemodynamical conditions), while accounting for mechanical deformations in the ocular tissues (biomechanical conditions) (Sala et al., 2018a,b; Sala, 2019). Such level of complexity calls for a multiscale modeling approach, where simplified network-based models that capture the essential dynamical features of complex systems at relatively low computational cost are coupled with three-dimensional models that capture spatial heterogeneities within the tissues (Figure 2). Specifically, the OMVS simulates: (i) the biomechanical behavior of cornea, sclera, choroid, retina, and lamina cribrosa via systems of partial differential equations describing their linear elastic dynamics; (ii) the blood circulation in the retinal vasculature and the central retinal vessels via systems of ordinary differential equations describing fluid flow as an electric current in a circuit-based model; (iii) the blood perfusion in the lamina cribrosa via a system of partial differential equations describing its Darcy-like porous medium dynamics.

Any mathematical model, regardless of its complexity, includes simplifying assumptions that capture some essential features of the system dynamics while keeping its numerical solution manageable. For instance, the OMVS assumes that the central retinal vessels and the retinal venules passively deform upon changes in transmural pressure, which results from the difference between intravascular pressure and IOP for intraocular vascular segments, and between intravascular pressure and RLTp for retrobulbar vascular segments. The current OMVS version does not account for active changes in the diameter of retinal arterioles due to blood flow autoregulation, which could be included as proposed by the authors in other works (Arciero et al., 2013; Guidoboni et al., 2014b). In the context of lamina cribrosa and ocular tissue modeling, the OMVS assumes a linearly elastic behavior, even though more realistic descriptions of the non-linear, possibly viscoelastic behavior were proposed in the literature (Roberts and Liu, 2017; Roberts et al., 2018) and would be interesting to include. Despite these underlying assumptions, the OMVS model brings the novelty of connecting complex biomechanic and hemodynamic responses to clinically measurable quantities related to tissue displacement and blood flow velocity, while yielding relevant information on tissues that are not currently accessible via noninvasive measurements, thereby providing a flexible tool to complement clinical and experimental studies and deepen the current understanding of ocular physiology in health and disease. More details on the mathematical and computational aspects, as well as on the validation of OMVS can be found in Sala (2019). The computational framework is also available as a web-based app for training and research purposes (Sala, 2019).

The OMVS is used to simulate hemodynamical and biomechanical conditions in 5 virtual subjects, who will be referred to as S0, S1, S2, S3, and S4. The subjects differ for their levels of IOP, RLTp, and TLPd, as summarized in Table 1. We emphasize that (i) all the patients have the same systolic/diastolic blood pressures (SP/DP = 120/80 mmHg); (ii) S0 represents the healthy baseline, with IOP = 15 mmHg and RLTp = 7 mmHg; (iii) S0, S2, and S4 have similar TLPd, but the individual levels of IOP and RLTp are different; and (iv) S1 and S3 have very different TLPd values.

**Table 1:** Values of systolic blood pressure (SP), diastolic blood pressure (DP), intraocular pressure (IOP), retrolaminar tissue pressure (RLTp), and translaminar pressure difference (TLPd) for the 5 virtual subjects (S0, S1, S2, S3, S4) considered in this study.

**Parameter**	**S0**	**S1**	**S2**	**S3**	**S4**	**Unit**
SP	120	120	120	120	120	mmHg
DP	80	80	80	80	80	mmHg
IOP	15	11	17	17	11	mmHg
RLTp	7	10	10	3	3	mmHg
TLPd	8	1	7	14	8	mmHg

**Figure 3:**
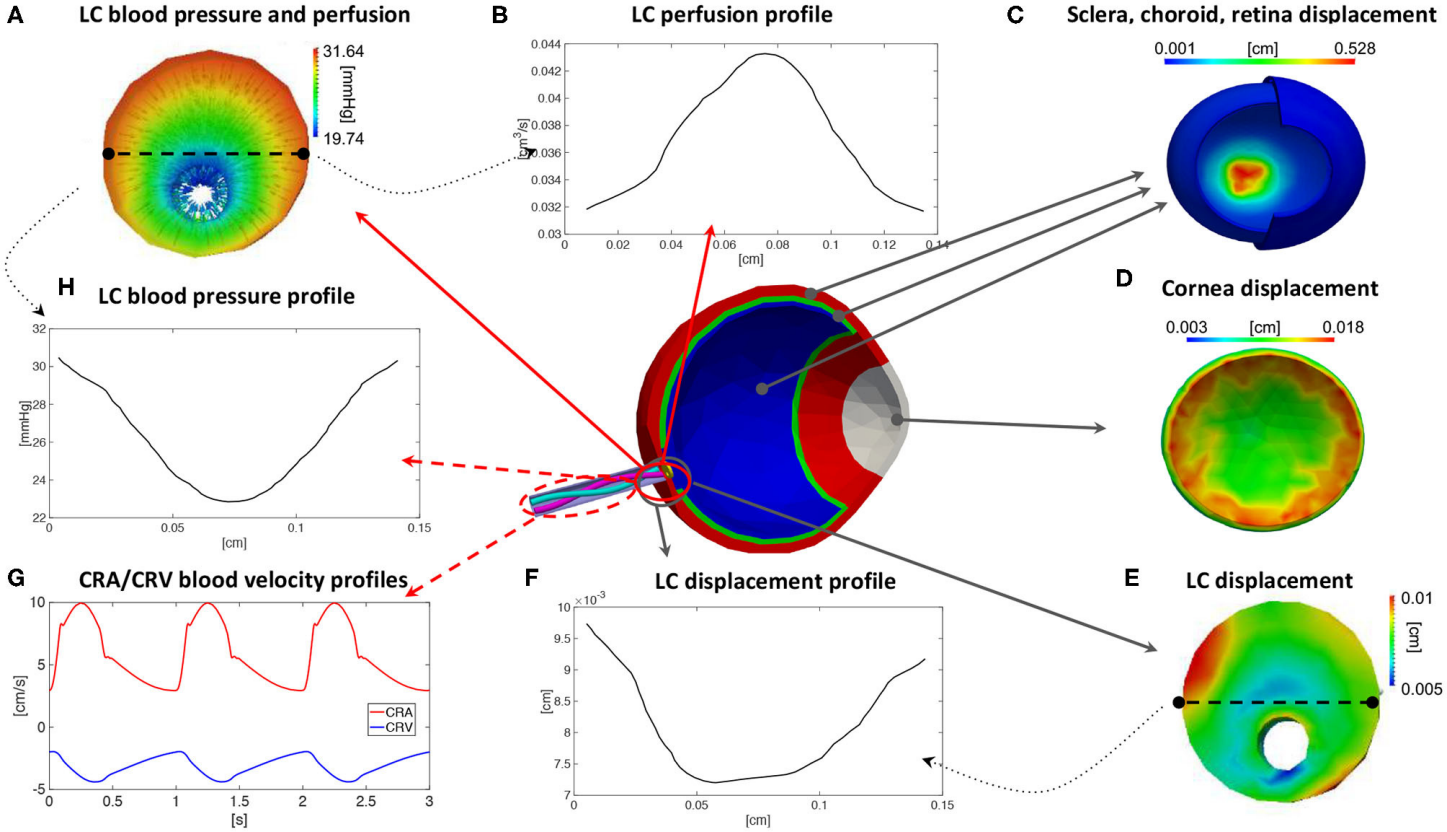
Illustrative schematic of the outputs that can be predicted by means of the Ocular Mathematical Virtual Simulator (OMVS) (Sala, 2019). The outputs include: quantitative three-dimensional colormaps of pressure, perfusion, and displacement fields in the lamina cribrosa **(A,E)**, with the possibility of plotting profiles along specific sections or lines **(B,F,H)**; quantitative three-dimensional colormaps of distributions of displacement in the ocular tissues **(C,D)**; time-dependent velocity waveforms in the central artery and vein **(G)**. LC, lamina cribrosa; CRA, central retinal artery; CRV, central retinal vein.

By utilizing the values in Table 1 as individualized inputs, the OMVS can be used to predict, for each virtual subject, (i) blood velocity waveforms in the pre- and post-laminar segments of the central retinal artery (CRA) and the central retinal vein (CRV); (ii) perfusion velocity and pressure within the lamina cribrosa; and (iii) the mechanical deformation of ocular tissues, including lamina cribrosa, sclera, and cornea. An illustrative schematic of the model outputs is provided in Figure 3. It is important to emphasize that some of the model outputs can be actually measured experimentally, such as the blood velocity in the CRA and CRV, see Figure 3G, and the deformations of cornea, sclera, and lamina, see Figures 3C–E, whereas the hemodynamic conditions within the tissue of the lamina cribrosa, see Figures 3A,B,H, are thought to be of high relevance for several diseases, such as glaucoma, but cannot be assessed with the technologies currently available.

The results reported in Figure 3 correspond to the virtual subject S0 representing the healthy baseline. Specifically, Figure 3G shows the model predicted waveforms for the blood velocity in the post-laminar segments of the CRA and CRV. It is interesting to notice that the CRV blood velocity has a negative sign, which is due to the fact that the blood flows in the opposite direction with respect to the flow in the CRA. During one cardiac cycle, the magnitude of the blood velocity waveforms are predicted to oscillate between 3 and 10 cm/s for the CRA and between 2 and 4.5 cm/s for the CRV. These results are consistent with measurements obtained via Color Doppler Imaging (Kaiser et al., 1996; Dellafiore et al., 2015; Cutolo et al., 2016; Kimyon et al., 2017). For example, the study by Kaiser et al. involving 189 healthy adult volunteers found that the CRA velocity varied between a systolic peak of 11.0 ± 1.8 cm/s and a diastolic minimum of 3.3 ± 0.9 cm/s, whereas the waveform in the CRV varied between a minimal value of 3.3 ± 0.7 cm/s and a maximal value of 4.5 ± 0.9 cm/s (Kaiser et al., 1996). Figure 3E shows the model predicted displacement of the lamina cribrosa with respect to the unloaded reference configuration. The colormap shows that the displacement is not uniformly distributed. When observing its profile along a particular direction across the lamina, see Figure 3F, the displacement exhibits a maximum difference between the central and peripheral regions of approximately 25 μm, which is consistent with the range of 59±64 μm reported in control eyes by Yan et al. (1994). Furthermore, the colormap for the scleral displacement in Figure 3C shows a larger deformability at the level of the optic nerve head, consistently with the study in Myers et al. (2010), and the colormap for the corneal displacement Figure 3D shows a range between 0.003 and 0.18 cm, which is consistent with that reported in Elsheikh et al. (2007).

The good agreement between model predictions and published experimental and clinical data for multiple measurable variables pertaining to ocular hemodynamics and biomechanics in healthy conditions, represented by the virtual subject S0, provide confidence in the model predictions pertaining to altered situations, represented by the four virtual subjects S1, S2, S3, and S4. Specifically, the results of the OMVS simulations for four virtual subjects (S1, S2, S3, S4) are summarized in Table 2 in terms of maximum percent differences in selected outputs of clinical interest with respect to the baseline subject S0. For the blood velocity in the pre- and post-laminar segments of the CRA and CRV, we calculated the maximum percent differences between the whole waveform over a cardiac cycle, see Figure 3H. For the perfusion velocity and displacement within the lamina cribrosa, we calculated the maximum percent differences along a selected cross section at each time instant, see Figures 3B,F, and then took the maximum over a cardiac cycle. Interestingly, the simulation results predict that subjects with similar TLPd (see S2 and S4) would exhibit similar biomechanical displacements of the lamina cribrosa tissue, with changes of <5% with respect to baseline, but very different hemodynamic conditions in the central retinal vessels, with the largest changes occurring in the CRA or in the CRV depending on whether the individual values of IOP and RLTp are lower or higher than their baseline values. Furthermore, in individuals with different TLPd values (see S1 and S3), the lamina cribrosa may exhibit changes of similar magnitude but different direction in the lamina displacement, which may be accompanied by hemodynamic changes in the CRA or in CRA depending on whether TLPd is lower or higher than its baseline value.

**Table 2:** Summary of the maximum values of percent differences with respect to baseline in outputs of clinical interest for four virtual subjects as predicted by the Ocular Mathematical Virtual Simulator (OMVS).

**Outputs of interest**		**Virtual subjects**			
**Anatomical site**	**Biophysical variable**	**S1 (%)**	**S2 (%)**	**S3 (%)**	**S4 (%)**
CRA pre-laminar	Blood velocity	7.93	−0.99	−0.33	14.26
CRA post-laminar	Blood velocity	8.17	−0.5	0.09	14.52
CRV pre-laminar	Blood velocity	0.77	11.66	0.75	1.06
CRV post-laminar	Blood velocity	3.22	12.89	40.87	3.57
Lamina cribrosa	Perfusion	−1.97	3.18	0.25	−5.36
Lamina cribrosa	Displacement	26.67	4.67	−22.67	−1.33

Interestingly, the predicted changes in the perfusion of the lamina cribrosa remain always <6% with respect to baseline. Given the fact that direct measurements of laminar perfusion are not currently available, it is not possible at this point to determine whether or not the magnitude of the predicted changes is significant for the disease process. However, it is worth noticing that the most marked decrease in perfusion is predicted for the virtual subject S4, who is characterized by the lowest IOP and RLTp levels among the five considered subjects. These results seem to suggest that low RLTp levels, which are indicative of low CSF pressure, may leave the optic nerve head tissue, where the lamina cribrosa is located, more susceptible to ischemic damage, especially in conditions of low IOP. This hypothesis has been formulated as a result of the model simulations and, hopefully, will inspire the design of novel studies to test it experimentally.

It is important to note that ocular biomarkers exhibit a physiological variability and this makes it challenging to ascertain the quantitative significance of the percent changes reported in Table 2 in absolute terms. For example, deviations of approximately 1.9 and 0.7 cm/s are reported for CRA peak-systolic and end-diastolic velocities, and 1.05 and 1.61 cm/s for the CRV minimal and maximal velocities, respectively (Tobe et al., 2015; Kimyon et al., 2017). Furthermore, the physiological variability of the lamina cribrosa perfusion has yet to be established. Thus, the most important contribution of the results reported in Table 2 is the identification of trends and relative impact of changes in important factors (SP, DP, IOP, RLTp, TLPd) that are difficult to disentagle in clinical and experimental settings.

Overall, the virtual clinical test performed using the OMVS suggests that: (i) similar TLPd values may lead to similar biomechanical deformations but different hemodynamic conditions (see S2 and S4); (ii) elevated IOP influences the blood circulation in the CRV (see S2 and S3); (iii) different TLPd values may lead to different biomechanical and hemodynamic conditions depending on the individual values of IOP and RLTp (see S1 and S3). These quantitative insights on the complex relationship between biomechanical and hemodynamic conditions and the levels of IOP, RLTp, and TLPd could be used to optimize the design of novel clinical and experimental studies and expedite the development of novel methods to interpret ocular measurements as biomarkers for systemic conditions affecting the eye and the brain.

**Figure 4:**
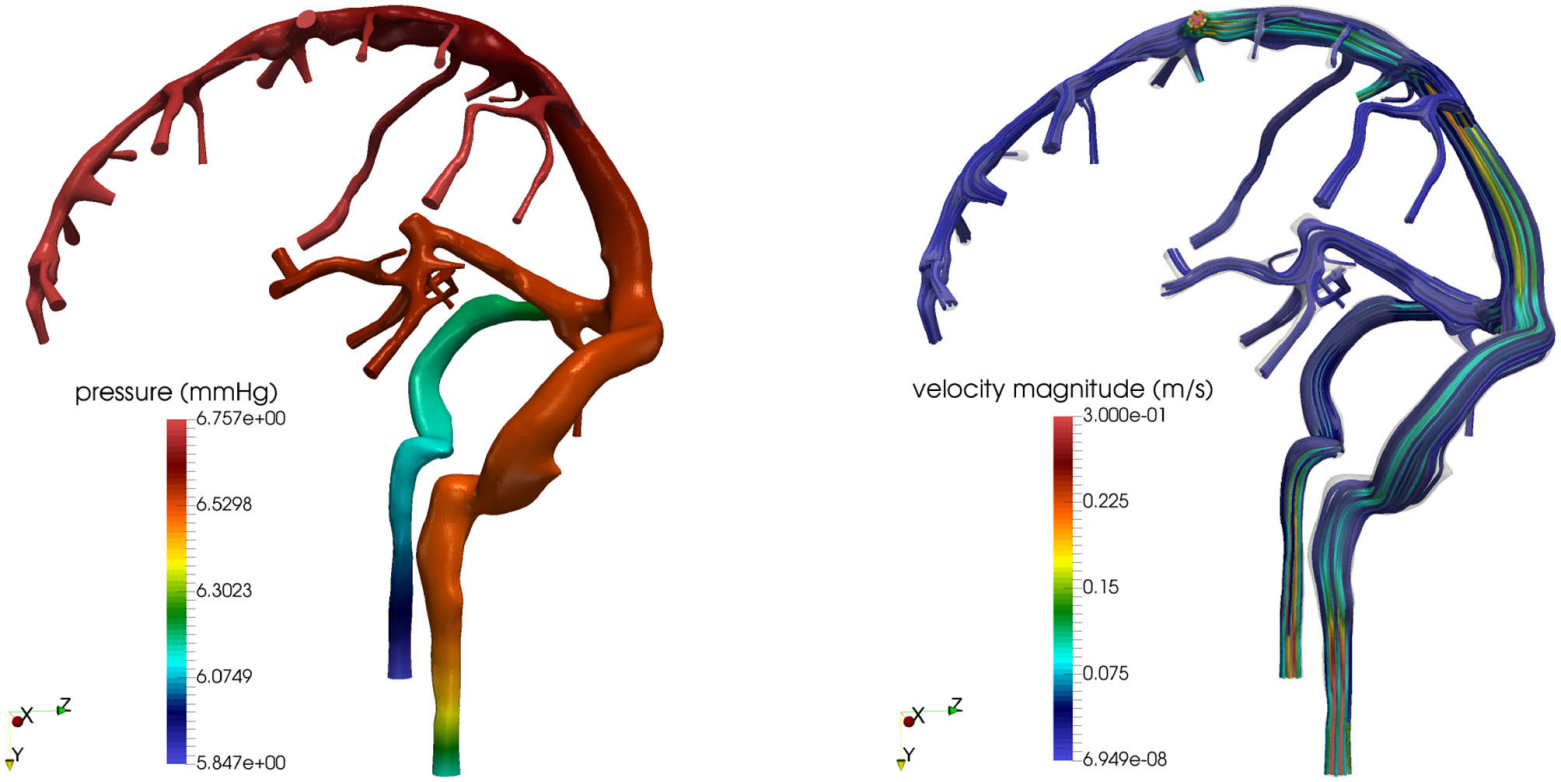
Cerebral venous hemodynamics. **(Left)** Pressure field. **(Right)** Streamlines. Images reproduced from Bertoluzza et al. (2017), with permission.

We remark that the virtual study illustrated in this section is by no means exhaustive, as it only serves the purpose of providing a general idea of the potential use of mechanism-driven models in the study of the eye-brain connections. Importantly, the multiscale/multiphysics platform provided by OMVS could be further extended to quantitatively explore additional hypotheses on pathogenic mechanisms, such as the role of elastic and viscoelastic properties of the ocular tissues (Bociu et al., 2016, 2019; Verri et al., 2018) and the effect of microgravity (Salerni et al., 2019). Furthermore, the circuit-based model of the fluid-dynamical connections between the eye and the brain proposed in Salerni et al. (2019) could be used to couple the three-dimensional model of the eye shown in Figure 2 with three-dimensional models of the brain such as those shown in Figure 4. Remarkably, the simulations reported in Figure 4 are based on the first three-dimensional sound mathematical and computational model for the venous part of the venous cerebral network proposed in Miraucourt et al. (2017), which complements previous works on the arterial side and on reduced models for the same compartment. Additionally, the modeling framework has been structured as to include uncertainties in the model parameters and realistic geometries reconstructed from images obtained via magnetic resonance angiographies (Chabannes et al., 2015; Passat et al., 2016).

## 7. Perspectives

NDD biomarkers based upon noninvasive ocular measurements could have a significant positive impact on many people world-wide. The example illustrated in section 6 has provided evidence suggesting that (i) the interpretation of ocular measurements as biomarkers for the brain status must account for patient-specific conditions that are local to the eye, such as the level of IOP, or systemic, such as the level of CSF pressure that influences RLTp; and (ii) mechanism-driven modeling provides a quantitative tool to enable such personalized interpretation. It should be also emphasized, though, that the predictions obtained via mechanism-driven models are subject to many limitations, such as the simplifying assumptions that need to be made on the anatomical and physiological features to include in the model and the availability of appropriate instruments to acquire the data to be used for the model. For example, the value of RLTp, which is an important model input, cannot be measured in a clinical settings with current technologies. Thus, at the present time, the value of RLTp is estimated from CSF pressure measurements which, however, are mostly focused on the lumbar segment of the spinal cord and intracranial compartments; whether and to what extent these measurements are indicative of the CSF pressure within the subarachnoid space surrounding the optic nerve remains a matter of debate (Januleviciene and Siaudvytyte, 2019).

Even though theoretical models are available to address specific biophysical issues of eye and brain, a single computational platform capable to treat the eye, the brain, and their connections in their entire complexity is not yet available, even though recently many scientists have urged the community to move forward in this unexplored and yet very promising direction (Frost et al., 2013; London et al., 2013; Jindal, 2015). Such an endeavor calls for significant advances in mathematical and computational sciences, where multiscale and multiphysics modeling are still active areas of research, with a parallel development of innovative technological solutions to accurately measure the quantities that are needed to characterize model inputs, calibrate model parameters, and validate model outputs. Furthermore, a large scale clinical study is warranted that is specifically designed to simultaneously collect measurements in the eye and brain of NDD patients and controls. Data from this comprehensive study could be used in synergy with mechanism-driven models with the goal of devising solid, validated, quantitative, predictive tools for the identification and interpretation of ocular biomarkers for cerebral conditions. Ultimately, the outcomes of such a multidisciplinary approach would set the foundation for novel paradigms in NDD diagnosis and clinical management, where noninvasive ocular measurements are utilized as biomarkers for cerebral fluid-dynamical and metabolic functions.

## Author Contributions

All authors listed have made a substantial, direct and intellectual contribution to the work, and approved it for publication.

## Conflict of Interest

GG would like to disclose that she receives remuneration for serving as a consultant for Foresite Healthcare LLC. AH would like to disclose that he received remuneration from Adom, Qlaris, and Luseed for serving as a consultant, and he serves on the board of Adom and Phileas Pharma. AH also holds an ownership interest in AdOM, Luseed, Oxymap, Phileas Pharma, and QuLent. All relationships listed above are pursuant to Icahn School of Medicine's policy on outside activities. The remaining authors declare that the research was conducted in the absence of any commercial or financial relationships that could be construed as a potential conflict of interest.
